# Multisensory mental representation in covid-19 patients and the possibility of long-lasting gustatory and olfactory dysfunction in the CNS

**DOI:** 10.1038/s41598-022-11119-6

**Published:** 2022-05-05

**Authors:** Barbara Tomasino, Gaia Pellitteri, Francesco Bax, Alessandro Marini, Andrea Surcinelli, Gian Luigi Gigli, Mariarosaria Valente

**Affiliations:** 1 Scientific Institute IRCCS “Eugenio Medea”, Polo Regionale del Friuli Venezia Giulia, Via della Bontà, 7, 33078 San Vito Al Tagliamento, PN Italy; 2 Clinical Neurology, Azienda Sanitaria Universitaria Friuli Centrale, Udine, Italy; 3 Neurology Unit, Department of Medicine (DAME), University of Udine, Udine, Italy

**Keywords:** Neuroscience, Psychology, Neurology, Signs and symptoms

## Abstract

Gustatory (GD) and olfactory (OD) dysfunctions are the most frequent neurological manifestations of COVID-19. We used mental imagery as an experimental psychological paradigm to access olfactory and gustatory brain representations in 80 Italian COVID-19 adult patients (68.75% reported both OD and GD). COVID-19 patients with OD + GD have a significantly and selectively decreased vividness of odor and taste imagery, indicating that COVID-19 has an effect on their chemosensory mental representations. OD + GD length and type influenced the status of mental chemosensory representations. OD + GD were become all COVID-19 negative at the time of testing. Data suggest that patients are not explicitly aware of long-term altered chemosensory processing. However, differences emerge when their chemosensory function is implicitly assessed using self-ratings. Among patients developing OD + GD, self-ratings of chemosensory function (taste, flavor) were significantly lower as compared to those who did not. At the level of mental representation, such differences can be further detected, in terms of a reduced ability to mentally activate an odor or taste mental image. Our study shows that COVID-19 infection not only frequently causes hyposmia and dysgeusia, but that may also alter the mental representations responsible for olfactory and gustatory perception.

## Introduction

In the current pandemic, COVID-19 patients have increasingly come to neurologists’ attention. This was not only for severe neurological complications, such as stroke, Guillain Barré Syndrome, epileptic seizures, confusion, and encephalopathy^1^, but also for mild to moderate neurological symptoms, predominantly in the absence of respiratory symptoms, e.g.^2–6^. Olfactory (OD) or gustatory (GD) dysfunctions have recently been recognized as a hallmark of COVID-19^7,8^. This observation is in line with findings from several countries e.g.^7–11^.

OD + GD data have been mainly collected from a self-reports or interviews^4,5,7–13^. Sensory alteration is not all-or-nothing. In a group study, for instance, dysfunction was total in 25% of the patients, strong in 33%, moderate in 27%, mild in 13% and normal in 2% of the patients^14^.

Data on the onset of these symptoms are inconsistent. Data show that OD and GD could appear late in the course of infection (second-third week)^15^ or also on average 4.4 (± 1.9) days after the onset of infection^10^ or may even precede COVID-19 systemic symptoms^7,11^. OD and GD symptoms length vary with mean of 7.5 ± 3.2 days^12^, with complete resolution in 40% of patients after 7.4 ± 2.3 days from their onset^12^ or after 28 days^10^.

The anatomical correlates of these symptoms are still unclear. Whether OD and GD are a result of peripheral nervous system damage, or COVID-19 alters olfactory and gustatory brain representations is a matter of debated. Biopsy data in patients with viral infections and anosmia indicate a direct damage of the olfactory epithelium^16^. These studies clearly point to the peripheral nervous system as the lesion substrate of OD and/or GD in COVID-19 patients e.g.^15^. The olfactory bulb might be the door of entry of SARS-CoV-2 into the CNS^7^. Neuroimaging, neurophysiological and CNS examinations start reporting data on the presence of encephalopathy in COVID-19 patients, in favor of the view that OD and GD may be a consequence of a central damage^17,18^. The brain could become infected via the olfactory bulb or via the bloodstream. A recent study shows that the nasal epithelium contains cells with high ACE2 expression, which allow the entrance of COVID-19 virus^19^.

Experimental psychology offers a paradigm to access olfactory and gustatory brain representations, namely multisensory mental imagery. Sensory mental imagery is the ability to see with the mind’s eye^20^ in the absence of a real percept. Neuroimaging studies have shown that there is an imagination-perception parallelism^21–31^. For example, imagining hearing simple monotones activates the auditory cortex^32^, imagination of hand, foot and tongue movements activates the corresponding motor representations^24^, imagining tactile stimulations of the back of the hand activates the contralateral sensory cortex^33^. Lastly, imagining tastes^23^ and odors^21^ activates the primary gustatory and olfactory cortex. Imagination and perception share the same neural substrates. Indeed, lesions in the sensory representations can cause imagination deficits in the corresponding modality^25,28,29^.

We evaluated the temporal relationship between a recovered perception and status of mental imagery (research question 1). To do this, we used a questionnaire to rate the patients’ actual (i.e., at the time of testing) olfactory and gustatory abilities, in order to assess the status of their perception. The experimental hypothesis is that clinical recovery does not correspond to a full recovery of sensory abilities which can turn in imagery deficits (Hypothesis 1). Evidence for long sequele of COVID-19 has been reported in patients manifesting phantosmia, parosmia, phantogeusia and paragusia in patients with a long sequele of COVID-19^34^. Alternatively, we could find that clinical recovery correspond to a full recovery of sensory abilities.

In addition, in order to test the nature of the olfactory and gustatory perception deficits in SARS-CoV-2 infection, we tested sensory mental imagination (research question 2). Based on previous studies using the paradigm of mental imagination as access to corresponding mental representations^35^, we assumed that an altered performance in olfactory and gustatory imagination tasks would indicate an alteration in mental representations or in the processing of such information (Hypothesis 2). We compared COVID-19 patients who had reported OD/GD perception and COVID19 patients who did not show them during the entire course of their disease. Grounded on the parallelism between perception and imagination, patients with OD/GD would have low performance in olfactory and gustatory mental imagination tests, demonstrating that COVID-19 degraded olfactory and gustatory mental representations. Alternatively, a normal performance on imagery tests would indicate dissociation between perception and imagery, implying that COVID-19 patients with OD/GD had preserved olfactory and gustatory mental representations (Hypothesis 3).

## Methods

### Participants

The study was approved by the Ethics Committee of the Azienda Sanitaria Universitaria Friuli Centrale (Prot. N. 0016723/P/GEN/ARCS) and carried out in accordance with the 2013 Fortaleza version of the Helsinki Declaration and subsequent amendments. All subjects were informed about the aim of the study and gave their written informed consent prior to inclusion.

A sample of 113 (out of 130 contacted and who did not want to take part to the study) Italian adults among patients with a positive nasopharyngeal swab for SARS-CoV-2 infection at the Azienda Sanitaria Universitaria Friuli Centrale (ASUFC, Udine, Italy) was initially included in this study. Participants were split into two groups: patients who had OD + GD and patients who did not. In the first part of the study, before they were administered mental imagery questionnaires, participants completed a questionnaire to collect information about: (i) their age, gender, years of education, handedness, city of residence and occupation; (ii) smoking status (non-smokers or previous smokers and, in the latter case, time since smoking cessation; (iii) information on possible causes of exclusion (see section on exclusion criteria). We asked them about: (i) the date they had tested positive for COVID-19, and the date they were re-tested and were found to be negative; (ii) whether they had GD/OD, and, if so, whether they experienced a decrease, alteration or loss of sensations. Finally, in patients who had suffered of OD + GD, we asked if these symptoms were still present, or their duration, in case of recovery.

#### Inclusion and exclusion criteria

Subjects meeting the following criteria were included in the study: (i) positive test for COVID-19; (ii) age between 18 and 75; and (iii) informed consent to participate in the study.

**Figure 1:**
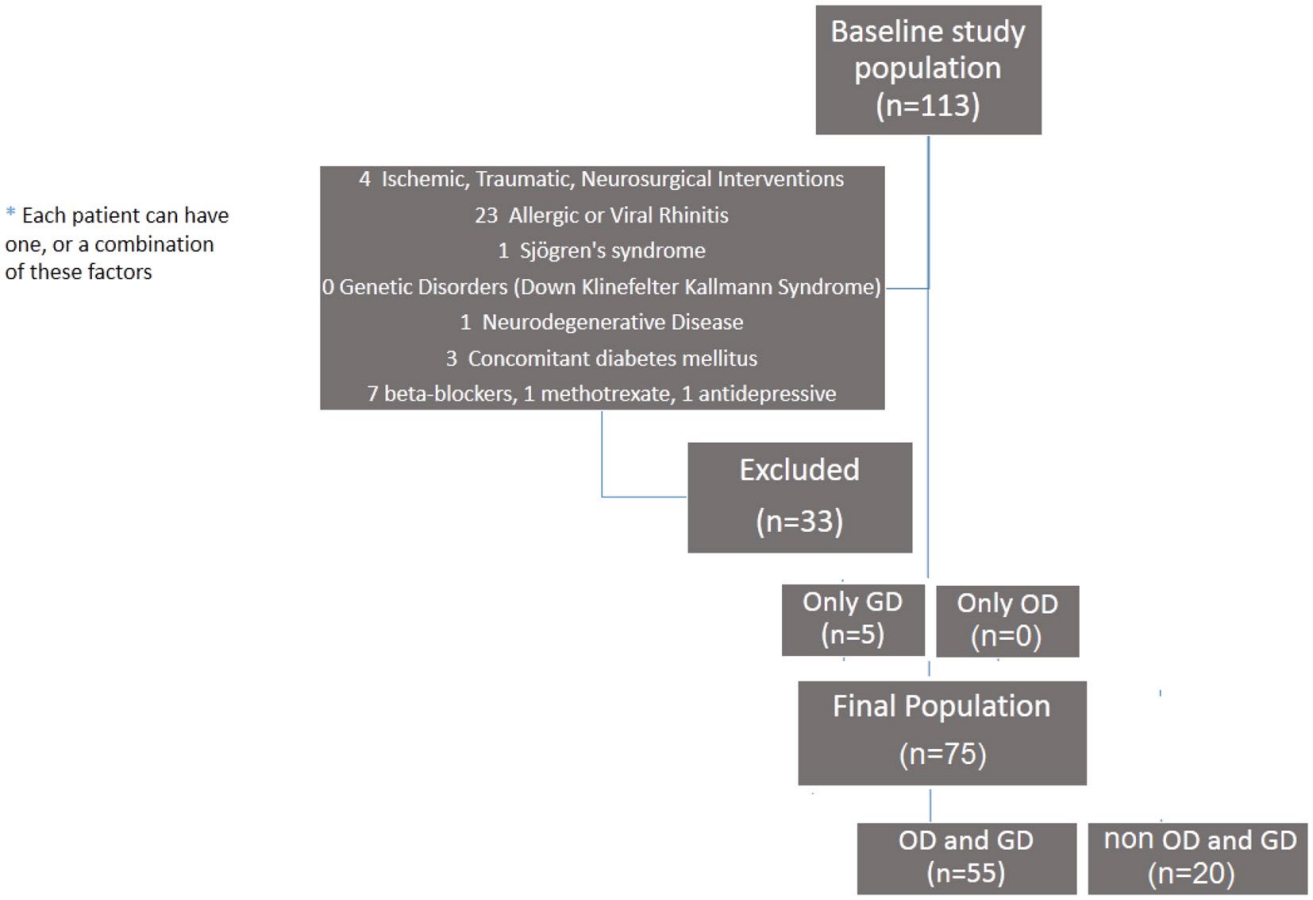
Patients included after the screening based on exclusion criteria.

Subjects meeting one or more of the following criteria were excluded from the study (see also Fig. 1): (i) known congenital structural anomalies of the Central Nervous System (CNS) such as cerebral or cerebrovascular malformations; (ii) known acquired structural lesions of the CNS, such as ischemic, traumatic events, brain tumors, in particular olfactory groove meningioma, or outcomes of neurosurgery interventions; (iii) known neurocognitive developmental deficits; (iv) conditions associated with changes in taste and smell, in particular allergic or viral rhinitis, exposure to organic solvents, HIV infections, Sjögren syndrome, states of malnutrition with zinc deficiency, neurodegenerative diseases (Alzheimer's disease, Huntington's chorea, Parkinson's disease, Pick's disease, multiple system atrophy), genetic diseases (Down, Klinefelter, and Kallmann syndromes); (v) concomitant diabetes mellitus; (vi) concomitant pharmacological therapies (especially: beta-blockers, calcium channel blockers, ACE inhibitors, anti-depressants, antibiotics, antifungals, methotrexate, chemotherapy); (vii) smoking behaviour.

### Paradigm and tasks

We used four questionnaires taken from the published literature and two additional experimental questionnaires developed specifically for the study. All questionnaires were self-assessment measures.

#### Self-assessment of taste and odor perception

The patients’ level of flavor, taste and odor perception at the time of testing was assessed by the following two ratings.

##### Self-assessment questionnaires, flavor and taste score^36^

Patients were asked to rate their current chemosensory function, namely flavor (“How would you rate your fine taste, e.g., during eating and drinking?”) and taste perception (“How would you rate your basic taste: sweet, sour, salty, bitter?”) by using a visual analogue scale ranging from 0 (“no taste/flavor perception”) to 10 (“excellent flavor/taste perception”; Cronbachs Alpha, calculated on our dataset was 0.923).

##### Self-assessment questionnaires, olfactory score^37^

Patients were asked to evaluate their sense of smell (“How would you rate your sense of smell?) by using a nine-point visual analogue scale ranging from 1 (“good sense of smell”) to 9 (“excellent sense of smell”).

#### Multisensory mental imagery vividness ratings

The following three questionnaires were used to measure the patients’ level of (i) general mental imagery vividness (Plymouth Sensory Imagery Questionnaire^38^); (ii) vividness of olfactory imagery for different entities, e.g. odors involving food or other perceptible odors in external or internal environments (Vividness of Olfactory Imagery Questionnaire—VOIQ^39^); (iii) vividness of olfactory and gustatory imagery for food and beverage (designed for the purposes of the present study).

##### Plymouth sensory imagery questionnaire—Psi-Q^38^

Psi-Q consists of 7 sub-tests, each including 5 items, one for each sensory modality (visual, auditory, olfactory, taste, touch, bodily sensation, and emotional feeling). A typical item is ‘Imagine the appearance of…’ (or ‘the sound of…’, ‘the taste of…’, ‘the odor of…’, and so on). Participants were asked to rate their image on a 10-point scale ranging from 0 (no image at all) to 10 (as vivid as real life). The Psi-Q allowed verifying the presence of dissociations between imagery in different sensory modalities (taste and/or olfaction vs. other sensory modalities). All items were summed to a total score, with low values reflecting good odor imagery abilities, and high values representing poor olfactory imagination abilities. In addition, total values were calculated for each category (Cronbachs Alpha of 0.91).

##### Vividness of olfactory imagery questionnaire—VOIQ^39^

VOIQ includes 16 odors belonging to four different categories: personal hygiene (bath), food-related (barbecue), tobacco and vehicles (car). For each category patients are presented with four sentences and asked to rate the vividness of their imagination on a five-point Likert scale (1 = perfectly realistic and as vivid as the real odor; 5 = No odor at all, you only “know” that you are thinking of an odor). All items were summed to a total score, with low values reflecting good odor imagery abilities, and high values representing poor olfactory imagination abilities. In addition, total values were calculated for each category (Split-half reliability 0.858).

##### Olfactory and taste mental imagery of food and beverages

We created a list of 40 words of food and drinks inspired by the list published in The FoodCast research image database^40^ presented in Appendix 1. Patients were explicitly asked to imagine the odor and evaluate the vividness of smell (“How would you rate your vividness of smell?) by using a 6-point visual analogue scale ranging from 0 (“No odor at all, you only “know” that you are thinking of an odor”) to 5 (“perfectly realistic and as vivid as the real odor”, Cronbachs Alpha, calculated on our dataset was 0.988).

Additionally, in a second questionnaire, they were explicitly asked to imagine the taste and to evaluate the vividness of taste (“How would you rate your vividness of taste?) by using a 11-point visual analogue scale ranging from 0 (“No taste at all, you only “know” that you are thinking of a taste”) to 10 (“perfectly realistic and as vivid as the real taste”, Cronbachs Alpha, calculated on our dataset was 0.987).

### Data acquisition

In addition to patients' responses to the above questionnaires, we collected demographic information, such as gender, age, area of residence, education and manual dominance^41^ were collected electronically. We also collected important data on patients’ current or previous smoke habits: these last factors, although not considered among the exclusion criteria, were taken into account during the process of data analysis, representing possible non infective causes of altered taste and smell.

### Sample size calculation

An a-priori analysis was conducted using G*Power^42^ to test differences between the means of two independent groups using t-test (two-tailed), a size average effect (d = 0.65), and the alpha of 0.05 and an expected ratio of 2:1 between the groups. Based on the results, a sample size of 86 participants was required to obtain a power of 0.80.

### Data analysis

Statistical analysis was performed using the Statistical Package for the Social Sciences (SPSS, Chicago, Illinois), version 20.0. The characteristics of the study population were described using means and standard deviation for continuous variables and percentages for categorical variables. Data was tested for normal distribution using the Shapiro–Wilk test. Variables did not fulfil requirements for parametric testing.

#### Statistics for research question 1

Subjective self-ratings for taste and odor perception were compared between the OD + GD vs the non OD + GD groups. Mann–Whitney *U*-test was used to compare continuous variables between groups. Comparison of categorical variables was performed by Fisher’s exact test, as appropriate.

Lastly, in order to analyze the relationship between variables, we choose an appropriate measure of association, using Kendall’s coefficient of rank correlation (if one variable was continuous and the other one was ordinal, or when both variables were ordinal), or, alternatively, point-biserial correlation coefficient (when one variable was continuous and the other was nominal, with two categories). For these correlation analyses we corrected for multiple comparisons by using Bonferroni correction.

#### Statistics for research question 2

Psi-Q, VOIQ, gustatory and olfactory mental imagery of food and beverages scores were compared between the OD + GD vs the non OD + GD groups.

Mann–Whitney *U*-test was used to compare continuous variables between groups. Comparison of categorical variables was performed by Fisher’s exact test, as appropriate.

Lastly, in order to analyze the relationship between variables, we choose an appropriate measure of association, using Kendall’s coefficient of rank correlation (if one variable was continuous and the other one was ordinal, or when both variables were ordinal), or, alternatively, point-biserial correlation coefficient (when one variable was continuous and the other was nominal, with two categories). For these correlation analyses we corrected for multiple comparisons by using Bonferroni correction.

## Results

### Population characteristics

All enrolled patients (n = 80) were tested positive for SARS-CoV-2 infection. They showed COVID-19 positivity periods which were quite homogeneous in terms of duration, for a mean of 30.43 ± 17.85 days. None of the patients was positive at the time of testing (performed at 151.44 ± 75.07 days from being found to be negative. During the acute phase of disease 68.75% had co-present OD + GD (only 5 patients had GD alone and were not entered in the analyses). The final sample included 75 patients (see Table 1 for their clinical details and Table 2 for their socio-demographic details) from northeastern Italy.

**Table 1:** Covid-19 symptom details in relation to OD + GD (olfactory and gustatory dysfunction).

Parameters	Value
Tested positive for COVID-19	80/80
Currently (at the time of the study) positive for COVID-19	0/80
Lenght of COVID-19 positivity (N = 80)	30.43 ± 17.85 days
Delta time of test—time of negativization (N = 80)	151.44 ± 75.07 days
Lenght of COVID-19 positivity (OD + GD patients)	28.5 ± 16.029 days
Delta time of test—time of negativization (OD + GD patients)	154.01 ± 89.02 days
OD + GD	55/80
Selective OD	0/80
Selective GD	5/80
Type of OD	Alteration 3/55 Reduction 19/55 Loss 33/55
Type of GD	Alteration 6/55 Reduction 21/55 Loss 28/55
Length of OD	Some weeks 29/55 Some days 17/55 Currently present 9/55
Length of GD	Some weeks 33/55 Some days 16/55 Currently present 5/55

**Table 2:** Patients’ with OD + GD socio-demographic details.

Parameters	Value
Number of patients	75
Gender	41 M; 34 F
Age	47.45 ± 13.96 yrs (range 18–71)
Education	15.47 ± 3.006 yrs (range 8–18)
Handedness	71 right-handed; 2 left-handed or ambidextrous
Occupation	17.33% health professional 2.6% restaurant staff 16% artisan/worker/technician/builder 12% teacher/educator/student 12% businessman/self-employed/agent 14.6% employed/designer 12% administrator/lawyer/executive/freelance/official 13.33% other

At the time of receiving our tests, 91.6% were no longer symptomatic for OD + GD. Nonetheless, some patients still had OD + GD (see Table 3). We re-run the analyses by excluding them.

**Table 3:** Tobacco smoking habits.

Parameters	Number of patients
Tobacco smoke	Never smoked 47/75 Former-smokers 28/75
Time since smoking cessation	> 5 years 16/28 2–3 years 4/28 3–6 months 4/28 1 month/2 weeks 4/28

Based on strict exclusion criteria, none of the patients had a previous relevant clinical and medical history and none was a smoker. We collected information about time since smoking cessation for additional analyses (see Table 3).

The OD + GD and non-OD + GD samples were comparable in terms of gender (30M/25F vs. 13M/12F, Fisher’s Exact Test p > 0.05., n.s.), smoking (39/12 vs. 12/8, Fisher’s Exact Test p > 0.05., n.s.), handedness (51/4 vs 20/0, Fisher’s Exact Test p > 0.05., n.s.), age (Mann–Whitney U, Z = − 0.95, p > 0.05, n.s.), education (Mann–Whitney U, Z = − 0.73, p > 0.05, n.s.), time since smoking cessation (Mann–Whitney U, Z = − 1.373, p > 0.05, n.s.), and delta time of test—time of negativization (Mann–Whitney U, Z = − 0.291, p > 0.05, n.s.). The two groups significantly differed in terms of length of COVID-19 positivity (Mann–Whitney U, Z = − 2.199, p > 0.05, n.s.) with longer positivity length for the non OD + GD group.

OD consisted mainly in odor perception loss (60% of the cases), followed by reduction (34.54%) and few had altered odor perception (5.45%). Similarly, GD mainly consisted of taste loss (50.91%), partially of taste reduction (31.18%) and taste alteration (10.91%) in a minority of cases (see Table 3).

OD lasted for some weeks in 52.72% of the patients, and some days in 30.9% of the patients. It was present at the time of testing in 16.36% of the patients. GD lasted some weeks in 60% of patients, and some days in 29.09% of the patients. It was present at the time of testing in 9.09% of the patients.

### Results for research question 1: flavor, taste and odor perception self-estimates

#### Flavor and taste and odor self-assessments

Results of flavor and taste and odor self-assessments are presented in Table 4. Despite having clinically recovered (see Table 3), OD + GD patients had significantly lower ratings of subjective chemosensory ratings. Their ratings for flavor were significantly lower compared to the non-OD + GD group (Z = − 4.039, p < 0.001). The OD + GD group had significantly lower ratings for taste compared to the non-OD + GD group (Z = − 4.294, p < 0.001). No significant difference was found for subjective odors ratings between the OD + GD groups vs. the non-OD + GD groups (Z = − 1.247, p > 0.05).

**Table 4:** COVID-19 patients’ performance according to whether they had olfactory and gustatory (OD + GD) dysfunctions or not.

	Response range	OD + GD	Non-OD + GD	P value
**Subjective chemosensory function**				
Self-assessment questionnaires, flavor and taste score				
Self-evaluated flavor	0 (poor)–10 (excellent)	7.74 ± 2.2	9.48 ± 1.09	0.003, effect size = 0.47
Self-evaluated taste	0 (poor)–10 (excellent)	7.309 ± 2.26	9.25 ± 1.21	0.001, effect size = 0.5
Self-assessment questionnaires, olfactory score				
Self-evaluated olfactory	1 (good)–9 (excellent)	4.89 ± 2.82	4.7 ± 3.62	> 0.05
**Multisensory mental imagery**				
Plymouth sensory imagery questionnaire				
Sum	0 (poor)–10 (excellent)	339.6 ± 36.46	363.1 ± 23.704	0.007, effect size = 0.28
Visual		9.47 ± 0.67	9.74 ± 0.42	> 0.05
Sound		9.25 ± 1.02	9.65 ± 0.51	> 0.05
Smell		8.29 ± 1.93	9.32 ± 0.84	0.031, effect size = 0.23
Taste		8.32 ± 1.52	9.12 ± 1.075	0.026, effect size = 0.24
Touch		9.08 ± 1.18	9.53 ± 0.77	> 0.05
Body		8.84 ± 1.61	9.53 ± 0.77	> 0.05
Emotion		8.87 ± 1.37	9.29 ± 1.09	> 0.05
Vividness of olfactory imagery questionnaire				
Sum	1 (excellent)–5 (poor)	38.72 ± 16.86	30.15 ± 16.54	0.037, effect size = 0.24
Item 1		2.43 ± 1.23	1.75 ± 0.91	0.043, effect size = 0.24
Item 2		2.42 ± 1.13	1.99 ± 1.25	> 0.05
Item 3		2.53 ± 1.28	1.93 ± 1.23	> 0.05
Item 4		2.29 ± 1.15	1.96 ± 1.055	> 0.05
Gustatory imagery for food and beverage items				
Total	0 (poor)–10 (excellent)	7.818 ± 2.09	8.91 ± 1.38	0.043, effect size = 0.24
Olfactory imagery for food and beverage items				
Total	0 (poor)–5 (excellent)	3.78 ± 1.04	4.32 ± 0.76	0.046, effect size = 0.23

Analyses run by excluding those patients who still had OD + GD confirmed the results. OD + GD ratings for flavor were significantly lower compared to the non-OD + GD group (Z = − 3.242, p < 0.001). The OD + GD group had significantly lower ratings for taste compared to the non-OD + GD group (Z = − 2.967, p < 0.001). No significant difference was found for subjective odors ratings between the OD + GD groups vs. the non-OD + GD groups (Z = − 0.120, p > 0.05).

Patients’ ratings significantly correlate (Bonferroni adjusted alpha levels, exact p-value = 000833) with GD length and with the type of GD (see Table 5 for correlation between COVID-19 clinical details and ratings)*.* In addition, some between-tasks correlations were found significant and are reported below.

**Table 5:** Results of the correlation analyses.

	Flavor and taste and odor self-assessments			Psi-Q		VOIQ	Gustatory and olfactory mental imagery of food and beverages	
	Flavor	Taste	Odors	Psi-Q_taste	Psi-Q_smell	VOIQ_sum	Gustatory	Olfactory
COVID-19 positivity length	*τ*_b_ = − 0.035, *p* = 0.688	*τ*_b_ = 0.028, *p* = 0.749	*τ*_b_ = 0.057, *p* = 0.499	*τ*_b_ = 0.005, *p* = 0.951	*τ*_b_ = 0.080, *p* = 0.342	*τ*_b_ = -0.003, *p* = 0.966	*τ*_b_ = 0.019, *p* = 0.815	*τ*_b_ = 0.068, *p* = 0.399
Delta time between testing and recovery	*τ*_b_ = 0.106, *p* = 0.222	*τ*_b_ = 0.055, *p* = 0.526	*τ*_b_ = − 0.064, *p* = 0.452	*τ*_b_ = 0.016, *p* = 0.844	*τ*_b_ = − 0.055, *p* = 0.514	*τ*_b_ = 0.070, *p* = 0.390	*τ*_b_ = − 0.070, *p* = 0.386	*τ*_b_ = − 0.074, *p* = 0.359
Being a non-smoker or ex-smoker	*r*_*s*_(71) = − 0.029, *p* = 0.810	*r*_*s*_(71) = − 0.002, *p* = 0.984	*r*_*s*_(71) = 0.179, *p* = 0.134	*r*_*s*_(71) = − 0.022, *p* = 0.853	*r*_*s*_(71) = 0.052, *p* = 0.665	*r*_*s*_(71) = 0.014, *p* = 0.909	*r*_*s*_(71) = − 0.051, *p* = 0.671	*r*_*s*_(71) = -0.016, *p* = 0.894
Time since smoking cessation	*τ*_b_ = − 0.078, *p* = 0.422	*τ*_b_ = − 0.095, *p* = 0.332	*τ*_b_ = 0.162, *p* = 0.09	*τ*_b_ = − 0.049, *p* = 0.6	*τ*_b_ = 0.049, *p* = 0.603	*τ*_b_ = − 0.060, *p* = 0.513	*τ*_b_ = − 0.039, *p* = 0.671	*τ*_b_ = -0.040, *p* = 0.659
GD length	*τ*_**b**_** = **− **0.371,** *p* < **0.000**	*τ*_**b**_** = **− **0.379,** *p* < **0.000**	n.e.	*τ*_b_ = − 0.119, *p* = 0.197	n.e.	n.e.	*τ*_b_ = − 0.168, *p* = 0.06	n.e.
OD length	n.e.	n.e.	*τ*_b_ = 0.085, *p* = 0.363	n.e.	*τ*_b_ = -0.105, *p* = 0.257	*τ*_**b**_** = 0.246,** *p* = **0.006**	n.e.	*τ*_b_ = -0.113, *p* = 0.205
Type of GD	*τ*_**b**_** = **− **0.246,** *p* < **0.001**	*τ*_**b**_** = **− **0.194,** *p* < **0.001**	n.e.	*τ*_b_ = − 0.170, *p* = 0.06	n.e.	n.e.	*τ*_b_ = − 0.117, *p* = 0.190	n.e.
Type of OD	n.e.	n.e.	*τ*_b_ = − 0.063, *p* = 0.508	n.e.	*τ*_b_ = − 0.176, *p* = 0.062	*τ*_b_ = 0.157, *p* = 0.083	n.e.	*τ*_b_ = -0.093, *p* = 0.306

Significant values are in bold.

n.e. = not executed because of no interest in the planned correlation analysis.

### Results for research question 2: multisensory mental imagery vividness ratings

#### Plymouth sensory imagery questionnaire (Psi-Q)

**Figure 2:**
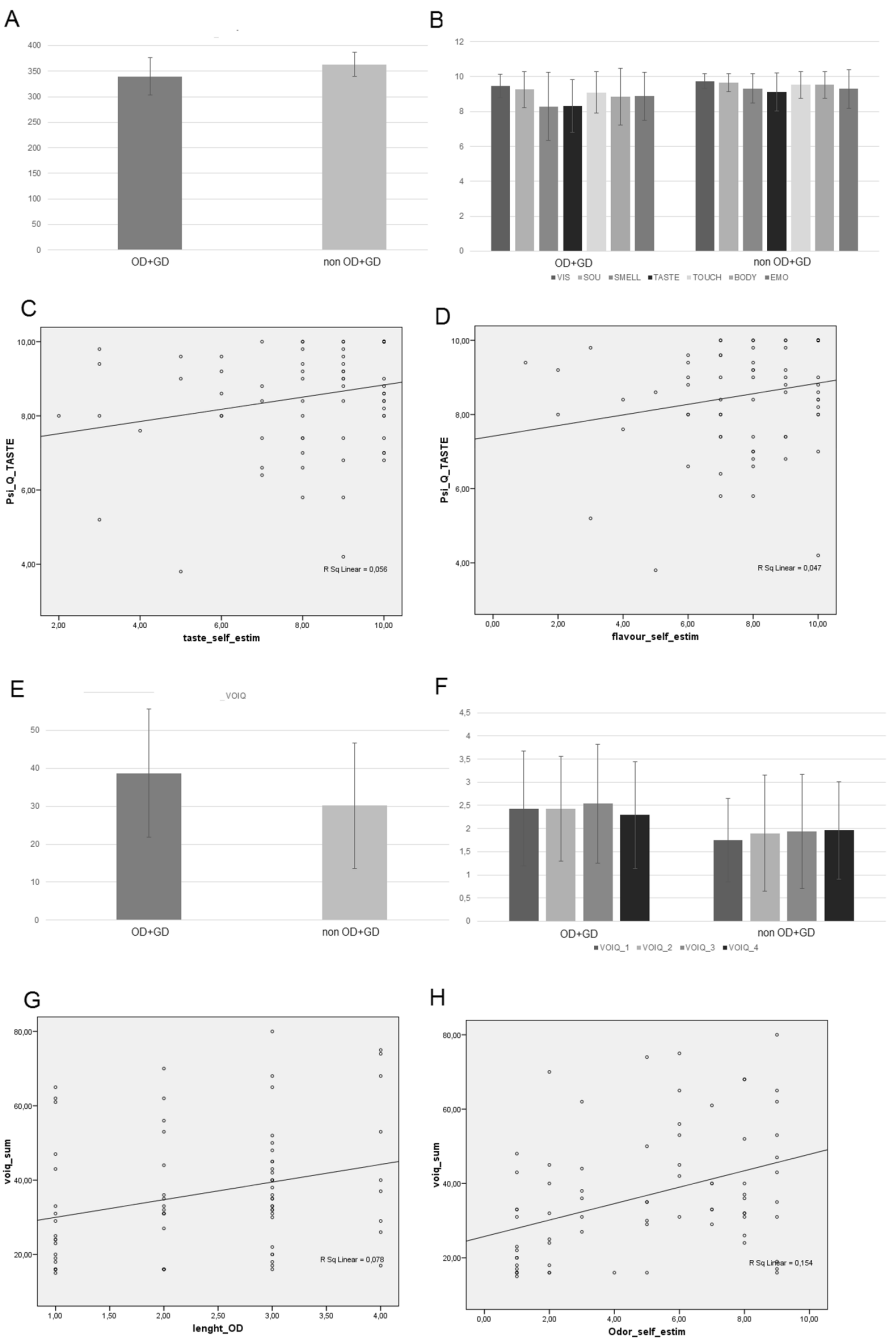
Patients’ performance at the Psi-Q sum of score (**A**), Psi-Q subtests (**B**), scale 0–10, poor-excellent, and correlation results (**C**, **D**). OD + GD = patients with odor and gustatory dysfunction. Patients’ performance at the VOIQ total score (**E**), VOIQ subtests (**F**), scale 1–5, excellent-poor, and correlation results (**G**, **H**). OD + GD = patients with odor/gustatory dysfunction.

Results of Psi-Q are presented in Fig. 2A and B. The OD + GD group significantly differed from the non-OD + GD group in total score (Z = − 2.37, p < 0.05), Psi-Q_taste (Z = − 2.099, p < 0.05) and Psi-Q_smell Z = − 1.971, p < 0.05), but not in visual (Z = − 1.254 n.s.), sound (Z = − 1.710 n.s.) touch (Z = − 1.368 n.s.), body (Z = − 1.346 n.s.) and emotion (Z = − 1.255, all p > 0.05. n.s.). Analyses run by excluding those patients who still had OD + GD confirmed the results (total score, Z = − 2.67, p < 0.007; Psi-Q_taste Z = − 2.229, p < 0.05; Psi-Q_smell Z = − 2.157, p < 0.05; visual, Z = − 1.544 n.s.; sound, Z = − 1.84 n.s.; touch, Z = − 1.96 n.s.; body, Z = − 1.81 n.s.; emotion, Z = − 1.51, all p > 0.05. n.s.).

Results of the correlation analyses (Bonferroni adjusted alpha levels, exact p-value = 0.0071) between clinical details and ratings showed no significant results (Table 5). In addition, between tasks correlations showed that Psi-Q_taste significantly correlated with subjective gustatory self-ratings (flavor, *τ*_b_ = 0.238, *p* = 0.007). Psi-Q_smell did not significantly correlate with subjective olfactory self-ratings (*τ*_b_ = − 0.108, *p* = 0.219, Fig. 2C,D).

#### Vividness of olfactory imagery questionnaire (VOIQ)

The OD + GD group significantly differed from the non-OD + GD group (total score, Z = − 2.095, p < 0.05, Fig. 2E,F). Analyses run by excluding those patients who still had OD + GD confirmed the results. The OD + GD group significantly differed from the non-OD + GD group, particularly on item 1 (Z = − 2.025, p < 0.05). All other items were comparable (item 2, Z = − 1.851; item 3, Z = − 1.633; item 4, Z = − 985, all p > 0.05. n.s.).

The total score (VOIQ_sum) significantly correlated (Bonferroni adjusted alpha levels, exact p-value = 0.00625) with OD length. In addition, between-tasks correlations showed that VOIQ_sum significantly correlated with subjective olfactory self-ratings (*τ*_b_ = 0.293, *p* = 0.001, see Fig. 2G,H), and with Psi-Q_smell (*τ*_b_ = − 0.307, *p* < 0.001).

#### Gustatory and Olfactory mental imagery of food and beverages

**Figure 3:**
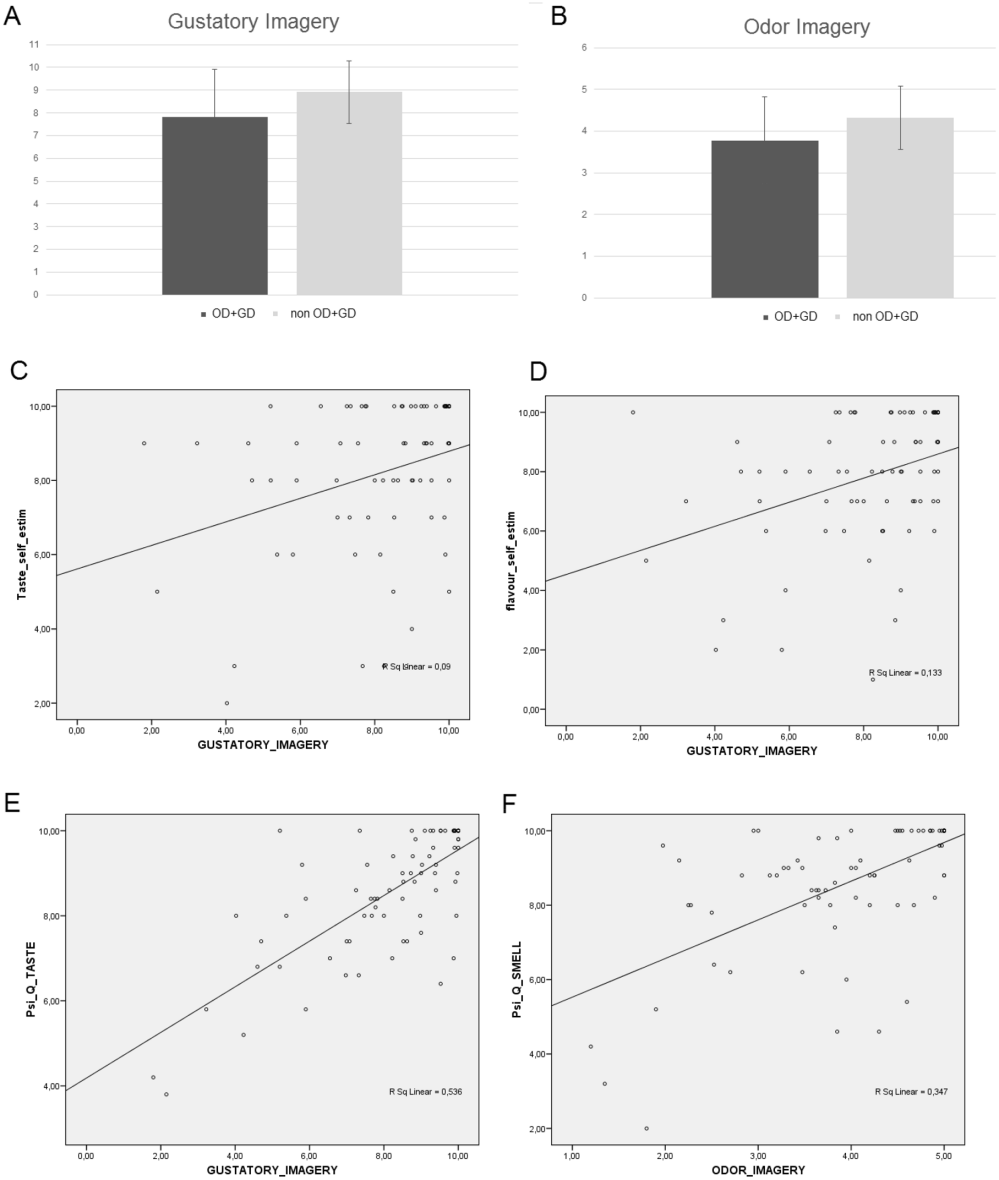
Gustatory imagery (**A**, scale 0–10, poor-excellent) and odor imagery for food and beverage (**B**, scale 0–5, poor-excellent) and correlation results (**C**–**F**). OD + GD = patients with odor/gustatory dysfunction.

The OD + GD group had significantly lower imagery vividness ratings of flavors compared to non-OD + GD group (Z = − 2, p < 0.05, Fig. 3A). Analyses run by excluding those patients who still had OD + GD confirmed the results. The OD + GD group had significantly lower imagery vividness ratings of flavors compared to non-OD + GD group (Z = − 2, p < 0.05).

The analysis testing between-tasks correlations showed that gustatory imagery vividness significantly correlated (Bonferroni adjusted alpha levels, exact p-value = 0.005) with subjective gustatory self-ratings (fine taste, *τ*_b_ = 0.293, *p* < 0.001; taste perception, *τ*_b_ = 0.244, *p* = 0.005), with Psi-Q_taste (*τ*_b_ = 0.523, *p* < 0.001) and with VOIQ_sum (*τ*_b_ = − 0.373, *p* < 0.001, Fig. 3C–E).

The OD + GD group had significantly lower odor imagery vividness ratings (Z = − 1.967, p < 0.05, Fig. 3B) compared to the non-OD + GD group. Analyses run by excluding those patients who still had OD + GD confirmed the results. The OD + GD group had significantly lower imagery vividness ratings of odors (Z = − 1.947, p < 0.05) compared to the non-OD + GD group. Between-tasks correlations showed that imagery vividness ratings of odors significantly correlated (Bonferroni adjusted alpha levels, exact p-value = 0.0055) with Psi-Q _smell (*τ*_b_ = 0.436, *p* < 0.001) and with VOIQ_sum (*τ*_b_ = − 0.360, *p* < 0.001, Fig. 3F). Imagery vividness ratings of odors did not correlate with subjective olfactory self-ratings (*τ*_b_ = − 0.133, *p* = 0.115).

## Discussion

The aim of this study was to explore whether OD/ GD in COVID-19 patients affected olfactory and gustatory mental imagery.

Firstly, we could reject the hypothesis that clinical recovery correspond to a full recovery of sensory abilities. Indeed, our main—certainly novel—finding is that, at the time of testing, OD + GD in post-COVID-19 patients (on average 154 days after they tested negative for COVID-19) was characterized by a significantly lower vividness of sensory mental imagery of odors and flavors as compared to patients who had not developed OD + GD. OD + GD patients reported that a deficit of smell/gustatory perception ceased; data showed that clinical recovery does not correspond to fully recovery of sensory abilities.

The timeline of disease course analysis showed that almost the entire population considered in our study had recovered from OD + GD at the time of testing, as emerged from patients’ clinical information forms filled out before the self-rating tests for chemosensory function and mental imagery vividness. Only few patients reported that OD + GD were still present.

However, signs of a not-yet-normal chemosensory processing were still present at the time of testing, on average 5 months after the COVID-19 swab had turned out to be negative. Indeed, patients’ self-ratings of chemosensory function (taste, flavor) were still significantly lower for those who had developed OD + GD compared to those who had not. Whether this pattern can be interpreted as long-term COVID-19 effects is unknown. However, data suggest that patients are not explicitly aware of a long-term altered chemosensory processing. Differences emerge only when they are implicitly assessed, through self-ratings of chemosensory function.

Secondly, we could reject the hypothesis that COVID-19 patients with OD/GD had preserved olfactory and gustatory mental representations. Indeed, at the level of mental representation, we further detected a reduced ability to mentally activate an odor or taste mental image.

The impact of COVID-19 on odor and taste mental representations expressed in COVID-19 patients who had developed OD + GD (our 55/80 patients presented both) have lower taste and odor mental imagery vividness. To the best of our knowledge, no study addressed sensory mental imagery in COVID-19 patients, thus there are no data in the literature on COVID-19 patients’ sensory mental representations. By contrast, at the level of chemosensory perception, many studies reported altered or loss of smell and taste^4,7,8^.

Mental imagery is used to access mental representations e.g.^35^. To rate the vividness of an imagined taste or odor, a mental representation of the corresponding perceptual experience has to be mentally re-enacted. A review of COVID-19 and chemical senses it is reported evidence that SARS-CoV-2 might alter cells and circuits involved in chemosensory processing and thereby change perception^43^. Our results add to previous evidence and indicate that COVID-19 may alter chemosensory processing related to taste and odor up to the level of the corresponding mental representations.

Results were consistent across questionnaires. The Psy_Q indicated that, across multisensory mental imagery, taste and odor sensory modalities showed a significant difference in vividness between OD + GD patients vs. non-OD + GD patients. The VOIQ evidenced that, within the smell sensory modality, overall OD + GD patients had significantly lower vividness compared to non-OD + GD patients. Lastly, we tested odor and gustatory imagery for food and beverage founding that OD + GD patients had significantly lower vividness compared to non-OD + GD patients. There is limited data in the literature about odor and taste imagery in patients with altered chemosensory perception. Decreased vividness of odor images can be found for anosmic patients^37^. The authors^37^ compared patients with peripheral olfactory dysfunction (ranging from hyposmia to anosmia) and healthy controls on the Vividness of Olfactory Imagery Questionnaire (VOIQ) and reported reduced mean vividness of odor images was decreased in anosmic patients. In hyposmic patients there was a trend towards significance for poorer odor imagery compared to controls.

Decreased olfactory imagery in patients with olfactory alteration, corresponds to lower activation in areas involved in olfactory processing. In an fMRI study^44^ on patients with olfactory loss who were presented with an olfactory imagery task, the authors reported that lower activation in olfactory brain regions was found in patients compared to healthy controls. Activation in primary gustatory and olfactory cortex is typically reported in functional imaging studies of mental imagery of tastes^23^ and odors^21^; for a meta-analysis see^45^. In healthy subjects, for example, the presentation of taste-related words^46^ caused a significantly stronger activation in primary and secondary gustatory cortices. In a PET study on odor imagery^21^, the authors found that olfactory imagery activates the piriform cortex, the orbitofrontal cortex and the insula. In another fMRI study^47^ in which participants were asked to smell and imagine pleasant and unpleasant odors and it was found that for both tasks (unpleasant vs. pleasant stimuli) triggered greater activity in the left frontal portion of the piriform cortex and the left insula. Taken together, these results show that patients with chemosensory alterations have a decreased vividness of olfactory imagery (less data is present in the literature about gustatory imagery in pathology) and lower activation in typical areas. Our results contribute new evidence to these studies including patients with various causes of olfactory loss^48^, showing that also COVID-19 pathology can affect mental chemosensory representations too.

Correlation analyses confirmed that alteration of perception and status of mental representations were closely related to each other. The longer OD + GD lasted, the lower the vividness of odor and taste mental imagery. Our results are consistent with those observed in a fMRI study^44^ on patients with olfactory loss, in which the length of olfactory dysfunction inversely correlated with activation in olfactory brain regions. By contrast, other authors^37^ did not report any correlation between disease length and vividness of olfactory imagery was obtained in patients with smell loss. We added new evidence, by showing that also length of COVID-19-related olfactory and/or gustatory dysfunction, too, can influence the status of mental chemosensory representations.

The vividness of odor and taste imagery also correlated with type of OD and GD. The more dysfunctional the chemosensory processing (from alteration to reduction to proper loss), the lower the vividness of odor and taste mental imagery. Our results are consistent with a previous study^37^, showing that anosmic vs. hyposmic patients had lower vividness of their mental odor images. Nonetheless, it should be acknowledged that the Self-assessment of taste and odor perception sometimes do not reflex the real olfactory and gustatory function of the patients, and this could represent in these cases a weakness of the study.

These data add new information in showing that the strength of COVID-19’s impact on chemosensory processing can differentially affect the corresponding sensory mental representations.

This applies to both odor and gustatory mental representations, for which we found no or very little data in the literature regarding to clinical populations. Findings were mainly from the neuroimaging literature.

There is nowadays increasing evidence in the literature on the association between COVID-19 and neurological manifestations^49^. Angiotensin-converting enzyme 2 (ACE2) receptor, which has been identified as the SARS-CoV-2 cellular receptor^50^, is widely expressed on the tongue, the oral cavity and the nasal mucosa^51,52^. Although the exact mechanisms are still unclear. Several authors have also suggested how SARS-CoV-2 may use ACE2 receptor binding to affect the neuroepithelium and spread to the olfactory bulb, entering the CNS, see the review by^53^. Moreover, ACE2 receptors are also expressed by the endothelial cells of the brain. It has been hypothesized that, once inside, SARS-CoV-2 mediated inflammatory cells recruitment and subsequent cytokine storm may lead to neuronal damage and death^53–55^. This could explain, on a speculative plan, COVID-19 induced stroke and encephalitis^56^. Indeed, a recent post-mortem study on a COVID-19 patient has showed the presence of the virus in neural and capillary endothelial cells of frontal lobe tissue^57^. Ultimately, there are first neuroimaging CT and MRI studies on patients with persistent COVID-19 induced anosmia that show scarcity of olfactory filia, olfactory bulb atrophy, and alterations of the primary olfactory cortex^58,59^. Our data are in line with previous studies showing evidence for long sequele of SARS-CoV-2 infection^34^.

We ascertained that the final patient group included in our study did not have any pathologies or clinical conditions, e.g.^60,61^ associated with changes in taste and smell. Patients were excluded if they took medications that could affect chemosensory perception e.g.^62^. Results were never significantly associated with being a no-smoker or an ex-smoker or with time since smoking cessation, e.g.^63^. Similarly, results were never significantly associated with COVID-19 positivity length and with time since patients ‘swab had turned out to be negative.

Future research can be focused on the study of OD + GD patients’ mental imagery abilities at follow-up, in order to measure time course of such impaired mental imagery. In addition, it would be worth performing an fMRI study measuring odor and taste mental imagery related activations.

### Limitation of the study

We acknowledge the lack of a matched healthy control group performing the same battery of questionnaires is a limitation of the present study, as we could have measured the amount of difference in terms of vividness between our patients and healthy controls.

## Conclusion

COVID-19 patients with OD + GD have a decreased vividness of odor and taste imagery, indicating that COVID-19 has an effect on patients’ chemosensory mental representations. It is held that mental imagery and perception share the same modality-specific representations. In this framework our results indicate the presence of a CNS dysfunction, which lasts much more than the duration of the infection. Our study confirms that OD + GD impact selectively on olfactory and gustatory imagery, but its results are not sufficient to indicate a permanent dysfunction of the primary sensory areas for smell and taste perception and cannot generalized to other sensory imagery modalities.

## Supplementary Information

Supplementary Information.

## Data Availability

The datasets analyzed for this study will be made available from the authors upon request.
