# Supplementary material for: Multisensory mental representation in covid-19 patients and the possibility of long-lasting gustatory and olfactory dysfunction in the CNS
Source: Sci Rep. 2022 May 5;12:7340. doi: 10.1038/s41598-022-11119-6 (PMC9069215; doi:10.1038/s41598-022-11119-6)
Supplement: Supplementary file 1 — Supplementary Information. [file 41598_2022_11119_MOESM1_ESM.doc]

**Appendix**

**List of food and beverage related words used in the Gustatory and Olfactory mental imagery of food and beverages task**

| **N item** | **Original Name (Italian)** | **English Name** | **Frequency *** | **Letters** | **Ingredients ^** |
| --- | --- | --- | --- | --- | --- |
| 1 | cracker | cracker | 2 | 7 | S |
| 2 | pane | bread | 164 | 4 | S |
| 3 | parmigiano | parmesian | 9 | 10 | A |
| 4 | gorgonzola | blue cheese | 1 | 10 | A |
| 5 | pizza | pizza | 20 | 5 | VSA |
| 6 | frittata | omelette | 17 | 8 | VA |
| 7 | miele | honey | 63 | 5 | S |
| 8 | torrone | nougat | 4 | 7 | SD |
| 9 | cioccolata | chocolate bar | 7 | 10 | D |
| 10 | bomboloni | cream donuts | 0 | 9 | D |
| 11 | olive | olives | 15 | 5 | V |
| 12 | salame | salami | 14 | 6 | M |
| 13 | hamburger | hamburger | 13 | 9 | VMS |
| 14 | mortadella | mortadella | 7 | 10 | M |
| 15 | würstel | frankfurters | 3 | 7 | M |
| 16 | arrosto | roasted chicken | 26 | 7 | M |
| 17 | origano | oregano | 2 | 7 | S |
| 18 | peperoncino | chilli pepper | 10 | 11 | V |
| 19 | cannella | cinnamon | 7 | 8 | S |
| 20 | liquirizia | licorice | 2 | 10 | D |
| 21 | lattuga | lettuce | 8 | 7 | V |
| 22 | prezzemolo | celery | 15 | 10 | V |
| 23 | cipolla | onion | 48 | 7 | V |
| 24 | mandarino | mandarin | 4 | 9 | F |
| 25 | ananas | pineapple | 15 | 6 | F |
| 26 | fragola | strawberry | 6 | 7 | F |
| 27 | pesca | peach | 98 | 5 | F |
| 28 | banana | banana | 12 | 6 | F |
| 29 | uva | grapes | 22 | 3 | F |
| 30 | mora | blackberry | 9 | 4 | F |
| 31 | vino bianco | white wine | 227 | 11 | B |
| 32 | coca-cola | coca-cola | 1 | 9 | B |
| 33 | camomilla | chamomile | 6 | 9 | B |
| 34 | aranciata | orangeade | 6 | 9 | B |
| 35 | limonata | lemonade | 5 | 8 | B |
| 36 | birra | beer | 60 | 5 | B |
| 37 | grappa | grappa | 14 | 6 | B |
| 38 | vino rosso | red wine | 227 | 10 | B |
| 39 | acqua | water | 945 | 5 | B |
| 40 | caffè | coffee |  | 5 | B |

* frequency with which the word occurs in the Italian language ^64^

^ V = Vegetable; F = Fruit; M = Meat; P = Fish; S = Seeds; D = Dessert; A = Animal derivative (i.e. eggs, dairy, cheese); B=beverage.
